# Signaling to Extracellular Signal-regulated Kinase from ErbB1 Kinase and Protein Kinase C: FEEDBACK, HETEROGENEITY, AND GATING*[Fn FN2]

**DOI:** 10.1074/jbc.M113.455345

**Published:** 2013-06-10

**Authors:** Rebecca M Perrett, Robert C. Fowkes, Christopher J. Caunt, Krasimira Tsaneva-Atanasova, Clive G. Bowsher, Craig A. McArdle

**Affiliations:** From the ‡ Laboratories for Integrative Neuroscience and Endocrinology, School of Clinical Sciences, University of Bristol, Whitson Street, Bristol BS13NY, United Kingdom,; § Endocrine Signaling Group, Royal Veterinary College, Royal College St., London NW10TU, United Kingdom,; ¶ Department of Biology and Biochemistry, University of Bath, Claverton Down, Bath BA27AY, United Kingdom,; ‖ Bristol Centre for Applied Nonlinear Mathematics, Department of Engineering Mathematics, University of Bristol, Bristol BS81TR, United Kingdom, and; ** School of Mathematics, University of Bristol, Bristol BS81TW, United Kingdom

**Keywords:** Cell Signaling, Epidermal Growth Factor (EGF), ERK, MAP Kinases (MAPKs), Protein Kinase C (PKC), ErbB1, Feedback

## Abstract

Many extracellular signals act via the Raf/MEK/ERK cascade in which kinetics, cell-cell variability, and sensitivity of the ERK response can all influence cell fate. Here we used automated microscopy to explore the effects of ERK-mediated negative feedback on these attributes in cells expressing endogenous ERK or ERK2-GFP reporters. We studied acute rather than chronic stimulation with either epidermal growth factor (ErbB1 activation) or phorbol 12,13-dibutyrate (PKC activation). In unstimulated cells, ERK-mediated negative feedback reduced the population-average and cell-cell variability of the level of activated ppERK and increased its robustness to changes in ERK expression. In stimulated cells, negative feedback (evident between 5 min and 4 h) also reduced average levels and variability of phosphorylated ERK (ppERK) without altering the “gradedness” or sensitivity of the response. Binning cells according to total ERK expression revealed, strikingly, that maximal ppERK responses initially occur at submaximal ERK levels and that this non-monotonic relationship changes to an increasing, monotonic one within 15 min. These phenomena occur in HeLa cells and MCF7 breast cancer cells and in the presence and absence of ERK-mediated negative feedback. They were best modeled assuming distributive (rather than processive) activation. Thus, we have uncovered a novel, time-dependent change in the relationship between total ERK and ppERK levels that persists without negative feedback. This change makes acute response kinetics dependent on ERK level and provides a “gating” or control mechanism in which the interplay between stimulus duration and the distribution of ERK expression across cells could modulate the proportion of cells that respond to stimulation.

## Teaser

**Background:** The mechanisms underlying acute ERK signaling are poorly understood.

**Results:** Feedback influences basal and acutely stimulated ERK responses but does not render signaling kinetics robust to ERK concentration.

**Conclusion:** Acute ERK response kinetics depend on ERK concentration and activation mechanism as well as feedback.

**Significance:** ERK responses to transient stimulation can be gated by ERK concentration, and short-term activation appears distributive rather than processive.

## Introduction

Many extracellular stimuli control cell fate via mitogen-activated protein kinase (MAPK) cascades (1–4). The best characterized of these is the Raf/MEK/ERK pathway, in which Raf kinases phosphorylate and activate MEK (MAPK/ERK kinase) that in turn phosphorylate ERK1 and ERK2 on Thr and Tyr residues of a Thr-Glu-Tyr activation loop (1–4). ERK (used here to denote ERK1 and/or ERK2) is mainly cytoplasmic in resting cells, but activation causes nuclear accumulation of dual phosphorylated ERK (ppERK)^3^ (5, 6), enabling phosphorylation of substrates that control transcriptional responses and, in turn, cell fate decisions (7, 8). MAPK modules show a broad range of input-output behaviors, reflecting the need for the wide range of MAPK stimuli to exert distinct effects on many cellular activities (8, 9). For example, stimuli causing sustained ERK activation and sustained translocation of ppERK to the nucleus can have different effects on biological outcome than those causing only transient activation (10–13), and these kinetic differences can reflect stimulus-specific feedback mechanisms. Indeed, ERK-mediated negative feedback can influence response kinetics rapidly (within 5–25 min) by inhibitory phosphorylation of Raf or Son of Sevenless (SOS) protein and more slowly (>40 min) by increasing expression of nuclear-inducible dual specificity phosphatases (14).

In addition to influencing response kinetics, ERK-mediated feedback can alter system sensitivity to changes in input (*i.e.* stimulus concentration) and to changes in system constraints and parameters (such as concentrations of network components and rate constants for their activation and inactivation) as well as cell-cell variability, all of which can be important for effects of ERK on cell fate (15, 16). Here, the “gradedness” of ERK signaling is of particular importance, as in many systems a gradual increase in stimulus causes graded responses in individual cells over a wide range of stimulus intensity, whereas in others there is an “ultrasensitive” response where large differences in output occur over a narrow input range, giving the appearance of an “all-or-nothing” response. Graded responses are thought to mediate reversible cellular activities, whereas all-or-nothing responses can impose a threshold for production of the binary decisions controlling irreversible processes such as cell cycle progression (17–22). In individual cells, graded inputs can drive digital outputs, and this analog-to-digital conversion can occur at different stages of a pathway. For example, in *Xenopus* oocytes increasing concentration of progesterone causes switch-like activation of ERK (23), whereas in Swiss 3T3 cells increasing EGF concentration causes graded activation of ERK with consequent switch-like stimulation of early gene expression and cell cycle progression (18). In this context the distributive activation of ERK is important; ERK binds MEK and is then monophosphorylated and released before rebinding to facilitate the second phosphorylation in the Thr-Glu-Tyr loop (24). This mechanism can result in ultrasensitivity of the Raf/MEK/ERK cascade (17). Despite this, graded responses are observed (17), and this may reflect scaffolding or molecular crowding, which promotes rapid enzyme substrate rebinding and thereby converts distributive to (pseudo)processive activation *in vivo* (25, 26). This is consistent with work on the yeast MAPK cascade where scaffolding of Ste11, Ste7, and Fus3 (MAPKKK, MAPKK, and MAPK, respectively) by Ste5 promotes graded signaling in response to stimulation with a mating pheromone (19). In that study the MAPK cascade could mediate graded or ultrasensitive responses, dependent upon the type of stimulus used (mating pheromone *versus* increased osmolarity). This fundamental feature of a single MAPK cascade mediating these distinct behaviors is also seen in T cells, where exposure to antigen-presenting cells elicits all-or-nothing ERK activation, whereas chemokine activation can cause graded responses (20).

The preceding discussion illustrates the richness of ERK signaling, with response kinetics, sensitivity, and cell-cell variability all having the potential to influence the consequences of ERK activation and all being subject to negative feedback. The importance of this is illustrated by the fact that ERK-mediated negative feedback dictates responsiveness of cells to inhibition of upstream kinases (21). However, most work on feedback control of this system has involved chronic (long term) stimulation, and less is known about its importance for regulation of the cascade under acute (short term) stimulation. Here, we have addressed this using automated cell imaging to monitor ERK phosphorylation and nuclear translocation as well as ERK-driven transcription in HeLa cells. We stimulated the cells with EGF to activate ErbB1 receptors or with phorbol 12,13-dibutyrate (PDBu) to activate protein kinase C (PKC). In unstimulated cells we found clear evidence that negative feedback influences population-averaged ppERK levels, cell-cell variability in ppERK levels, and system robustness. In stimulated cells negative feedback between 5 min and 4 h of stimulation with EGF or PDBu influenced variation and mean levels of ppERK, but we found no evidence for it affecting response sensitivity.

Previous work suggests that negative feedback could make the signaling system robust to changes in the concentrations of the proteins in the cascade (21), and we find clear evidence for this in unstimulated cells. However, when we explored relationships between total ERK and ppERK under short term stimulation, we observed maximal ppERK levels at submaximal ERK expression levels soon after stimulation (at 5 min) and then a switch to monotonic behavior within 15 min of stimulation. This occurred in two cell types (HeLa and MCF7 cells) and at a broad range of EGF concentrations in both the presence and absence of negative feedback. It could be modeled mathematically assuming distributive (but not processive) activation. Furthermore, increasing ERK expression levels increased the time taken to reach the maximal ppERK response, providing a mechanism for “gating” or control of the ERK response. Thus, in the non-equilibrium conditions of acute stimulation, there is a novel time-dependent change in the relationship between ERK and ppERK levels that persists in the presence of negative feedback, is suggestive of distributive activation, and makes acute ERK response kinetics dependent upon ERK expression levels in our system.

## EXPERIMENTAL PROCEDURES

### 

#### 

##### Cell Culture and Transfection

**FIGURE 1.**
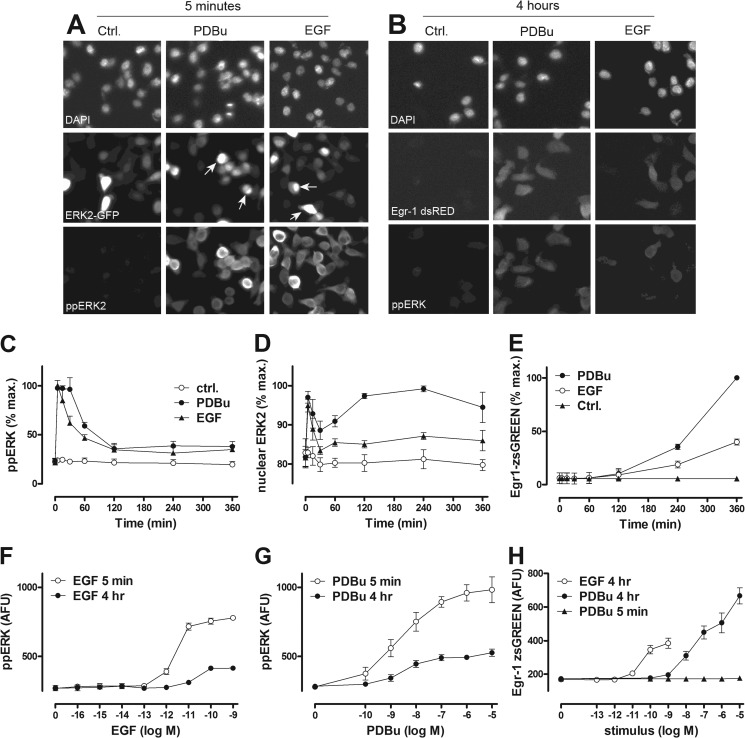
**Time and concentration dependence of EGF and PDBu effects on ERK in HeLa cells as revealed by semi-automated fluorescence microscopy.** The *top panels* are digital images illustrating the type of raw data used for this study. *Panel A* shows cells transfected with siRNA targeting endogenous ERK1 and ERK2, transduced with Ad ERK2-GFP, then stimulated for 5 min with or without EGF (10 nm) or PDBu (1 μm) before being fixed, stained, and imaged for DAPI (nuclei) and ERK2-GFP as well as ppERK2. *Panel B* shows cells transduced with Ad Egr1-dsRED and stimulated for 4 h with or without EGF or PDBu before being fixed, stained with DAPI, and imaged for DAPI, endogenous ppERK, and dsRED. Digital images were acquired for each fluorophore using a 10× objective and 6–10 fields of view per well, and automated image analysis was then used to define perimeters of nuclei and cells and fluorescence intensity within these areas, as described under “Experimental Procedures.” Each panel has a width of ∼170 μm and, therefore, shows ∼5% of the field captured. These are representative images (from the experiments used for *panels C–H*) illustrating the ability of EGF and PDBu to increase the proportion of ERK within the nucleus (*arrows*) and Egr1-driven transcription as well as whole cell ppERK levels. *Panels C* and *D* are time-courses from cells with endogenous ERK (*C*) or after siRNA-mediated knockdown of ERK and Ad-mediated add-back of ERK2-GFP (*D*) that were incubated from *t* = 0 with EGF (10 nm, *filled triangles*) or PDBu (1 μm, *filled circles*) or control medium (*Ctrl.*, *open circles*). *Panels F* and *G* are from cells with endogenous ERK that were stimulated 5 min or 4 h with the indicated concentrations of EGF or PDBu. *Panels E* and *H* are from cells transduced with Ad Egr1-zsGREEN and stimulated as shown. Cells were fixed, stained, imaged, and analyzed as above. The data are whole cell measures except for *D*, where the proportion of ERK2-GFP in the nucleus is shown. Data shown are population averages from 2–5 experiments (mean ± S.E., *n* = 2–5) and are in AFU or are normalized to maximal responses to PDBu (in each of the repeated experiments). Data obtained with the Egr1-dsRED reporter (*B*) are qualitatively similar to those with the Egr1-zsGREEN reporter (*E* and *H*).

HeLa cells and MCF7 cells (from European Collection of Cell Cultures) were cultured in 10% FCS-supplemented Dulbecco's modified Eagle's medium (DMEM). For experiments they were harvested by trypsinization and seeded at 3–5 × 10^3^ cells/well in 96-well plates. For some experiments ERK was knocked down by reverse transfection using RNAiMAX (Invitrogen) and two siRNA duplexes (Qiagen, Crawley, UK) each for ERK1 and ERK2 (27, 28). A mixture of all four ERK duplexes or control siRNA against GFP (Ambion, Warrington, UK) was used (at 2.5 nm total). Where ERK was knocked down, recombinant adenovirus (Ad) were used to add back previously characterized (29) imaging reporters consisting of wild-type (WT) ERK2 in tandem with green fluorescent protein (ERK2-GFP) or a catalytically inactive mutant (K52R ERK2-GFP). Cells were transduced with Ad ERK2-GFP in DMEM with 2% FCS 16 h after siRNA transfection. The Ad-containing medium was removed after 4–6 h and replaced with fresh DMEM with 0.1% FCS. For some experiments cells were transduced with Ad for an Egr-1 promoter driving expression of dsRED or zsGREEN. Ad5 zsGREEN and DsRedExpress vectors were made by digesting pzsGREEN1-DR and pDsRed-Express-DR (Clontech) with BamHI/NotI and subcloning fluorescent protein cDNAs into a corresponding digest of promoterless pAd5K-NpA vector (a gift from Prof. Beverly Davison, Gene Transfer Vector Core, University of Iowa). Egr-1 promoter was amplified using 5′-tat gta ctc gag acg gag gga ata gcc ttt cg-3′ forward primer and 5′-tat gta gaa ttc gag aac tga tgt tgg gtg gtg-3′ reverse primer using Egr-1-promoter-Luc vector (30) as template. The product was digested with XhoI and EcoRI (underlined) and subcloned into the corresponding digests of pAd5 zsGREEN1-DR and pAd5 DsRed-Express-DR. All Ad were generated from shuttle vectors as described (31) and were added to cells at the same time using 0.5–10 plaque-forming units (pfu)/nl. In the HeLa cell system used here this yields multiplicity of infection values of 10–100 and transduction efficiency approaching 100%. The cells were cultured for 16–24 h after transduction and were then stimulated with EGF or PDBu (Sigma) as outlined in the legends to Figs. 1 and 3–7. For some experiments the cells were also treated with a selective ERK inhibitor, FR180204 (Tocris Bioscience, Bristol, UK), or the structurally related negative control compound, 328008 (Calbiochem) as outlined in the figure legends.

##### High-content Image Acquisition and Analysis

Cells were cultured, plated, transfected, and transduced on Costar black-wall 96-well plates (Corning, Arlington, UK) as described (27, 29). After stimulation they were fixed in 4% paraformaldehyde (in PBS) and permeabilized in −20 °C methanol. In most experiments cells were stained for ppERK by blocking in 5% normal goat serum, PBS and probing with mouse anti-ppERK monoclonal antibody (MAPK-YT, 1:200, Sigma) in PBS and visualization with Alexa 488- or 546-conjugated goat anti-mouse antibodies (1:200 Invitrogen). Total ERK was stained with rabbit anti-ERK monoclonal (137F5, 1:100, Cell Signaling Technology, Hutchin, UK) and Alexa 546-conjugated goat anti-rabbit antibody (1:200, Invitrogen). Nuclei were also stained (600 nm DAPI in PBS), and for most experiments the fluorescence of ERK2-GFP, dsRED and/or zsGREEN was also visualized. Image acquisition was automated using an IN Cell Analyzer 1000 (GE Healthcare) microscope as described (32, 33) with a ×10 objective and excitation and emission filters of 360 and 460 nm for DAPI, 475 and 535 nm for Alexa 488, GFP, and zsGREEN, or 535 and 620 nm for Alexa 546 and dsRED. Representative digital images are shown in Fig. 1. Automated image analysis algorithms were used to define perimeters and fluorescence intensity within regions of interest (nucleus and cytoplasm or nucleus and nuclear collar). The multi-target analysis algorithm in the IN Cell Analyzer Work station was used to define nuclear perimeters from the DAPI stain. For cells without ERK2-GFP the fluorophores imaged could not be used as cell masks (because of low fluorescence in unstimulated cells) so we expanded the nuclear perimeter (3 μm collar) to capture cytoplasmic fluorescence. Whole cell (nucleus plus cytoplasm) fluorescence measures were used for ERK, ppERK, ERK2, ppERK2, dsRED, and zsGREEN and are reported in arbitrary fluorescence units (AFU). In some experiments the proportion of ERK2-GFP in the nucleus was calculated. For most experiments, replicate treatments were applied in 2–4 wells and 4–9 fields of view were collected per well, yielding data for >10,000 individual imaged cells (for each treatment in each experiment). This was used to produce population-averaged mean responses and for frequency distributions of individual cell measures (29) or was transformed and binned as described in the figure legends. Log concentration-response relationships were fitted (GraphPad Prism, La Jolla, CA) to Sigmoid curves of variable slope to estimate EC_50_ values and Hill coefficients.

##### Models and Modeling

The mathematical models were based on mass action kinetics using ordinary differential equations of the core ERK activation pathway mechanism. Two computational models, Processive and Distributive, were employed to investigate transient activation of ERK for different total ERK levels. Details are given in the supplemental data.

## RESULTS

### 

#### 

##### Negative Feedback Control of ERK Signaling

The Raf/MEK/ERK module is subject to rapid (transcription-independent) negative feedback by mechanisms including ERK-mediated phosphorylation of Raf and to slower (transcription-dependent) feedback by mechanisms including ERK-mediated induction of dual specificity phosphatases. In addition, different inputs to Raf (such as active ErbB1 or PKC) may have stimulus-specific upstream adaptive mechanisms. Such feedback has the potential to influence not only response kinetics but also cell-cell variability and response sensitivity and to do so in a time-dependent and stimulus-specific manner. Here we used automated microscopy (Fig. 1, *A* and *B*) to explore how ERK-mediated negative feedback influences ERK signaling in HeLa cells stimulated with EGF (ErbB1 activation) and PDBu (PKC activation). Anticipating that negative feedback could underlie desensitization we tested for this by monitoring the time-dependent effect of stimulation on ppERK levels.

As expected (27, 29), EGF and PDBu both caused rapid and transient ERK activation, increasing ppERK to the maxima at 5–15 min (Fig. 1*C*). These responses were initially comparable but were then more sustained with PDBu than with EGF. For some experiments we used siRNA to knock down endogenous ERK and recombinant Ad to add back ERK2-GFP. With this protocol, knockdown efficiency is >90%, and the reporter mirrors endogenous ERK activity (27, 29, 34). This again revealed more sustained elevation of ppERK with PDBu (not shown) and that both stimuli increased the proportion of ERK2 in the nucleus, effects that were again more sustained with PDBu (Fig. 1*D*). We also used these protocols to construct concentration-response curves at 5 min and 4 h. This revealed concentration-dependent increases in ppERK with responses lower at 4 h than at 5 min (Fig. 1, *F* and *G*). Curve-fitting revealed a time-dependent reduction in EGF potency, with EC_50_ values of 2 pm at 5 min and 15 pm at 4 h. The EC_50_ for PDBu (2.6 nm) and the Hill coefficients for EGF and PDBu (1.4 and 0.5, respectively) at 5 min were indistinguishable from those at 4 h (*p* > 0.1). For some experiments we also transduced cells with Ad Egr-1 zsGREEN or Ad Egr-1 dsRED as transcriptional readouts for ERK activation. Both stimuli caused increased zsGREEN (Fig. 1, *E* and *H*) from 1 to 2 h, with near linear accumulation from 2 to 6 h. Similar data were obtained after knockdown of endogenous ERK and add-back of ERK2-GFP with the Egr-1 dsRED reporter (not shown), and both responses were abolished by co-incubation with the MEK inhibitor PD184352 (10 μm), demonstrating MEK/ERK dependence (not shown). For each reporter, the PDBu effect was greater than that of EGF (Fig. 1, *E* and *H*, and data not shown). Together, these data confirm earlier work (27, 29, 33) showing that these ERK activation and translocation responses are more-or-less transient (Fig. 1, *C* and *D*) and that response kinetics typically differ for the two stimuli used. The data indicate the occurrence of negative feedback and suggest that some of its mechanisms are stimulus-specific. They also extend earlier work by showing that adaptation influences stimulus potency (Fig. 1, *F* and *G*) but not sensitivity (as measured by Hill coefficients at 5 min and 4 h). Moreover, they reveal that the transcriptional effect of PDBu is greater than that of EGF (Fig. 1, *E* and *H*), which most likely reflects the ability of PDBu to cause a more sustained phosphorylation and nuclear translocation of ERK (Fig. 1, *C* and *D*).

**FIGURE 2.**
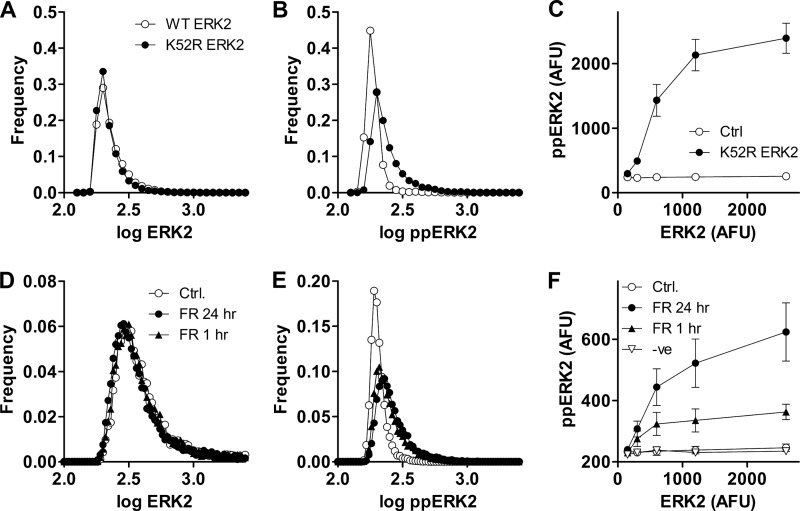
**In unstimulated HeLa cells ERK-mediated feedback reduces cell-cell variability in ppERK and increases system robustness to changes in ERK expression levels.** *Panels A* and *B* show data from experiments using siRNA to knock down endogenous ERK and Ad to add-back ERK2-GFP or K52R ERK2-GFP. Ads were used at 1 pfu/nl. The data are frequency-distribution plots of log ppERK2 or ERK2-GFP (in AFU) from single representative experiments each with >10,000 cells per plot and cells expressing either ERK2-GFP (*open circles*) or K52R ERK2-GFP (*filled circles*). Similar experiments were also undertaken with a higher Ad titer (10 pfu/nl), and pooling data from three such experiments revealed that CVs for ERK2-GFP expression were greater than those for ppERK2 (*i.e.* 0.038 ± 0.004 and 0.026 ± 0.002 at 1 pfu/nl) and that increasing the Ad ERK2-GFP increased cell-cell variability in ERK2-GFP (CVs 0.038 ± 0.004 and 0.104 ± 0.003 at 1 and 10 pfu/nl, respectively, *p* < 0.05) but did not increase variability for ppERK2-GFP (CVs 0.026 ± 0.002 and 0.028 ± 0.001 at 1 and 10 pfu/nl respectively, *p* > 0.1). CVs for ERK2-GFP did not differ between cells expressing WT- or K52R-ERK2-GFP (at either titer, *p* > 0.1), whereas the K52R mutation increased cell-cell variability in ppERK2-GFP measures at both titers (CVs increased from 0.026 ± 0.002 to 0.049 ± 0.003 at 1 pfu/nl (*p* < 0.05) and from 0.028 ± 0.001 to 0.098 ± 0.002 at 10 pfu/nl (*p* < 0.05)). *Panel C* was generated by binning individual cells according to their ERK2-GFP expression level and calculating the mean ppERK2 level in each bin. The bins had ranges of 100–200, 200–400, 400–800, 800–1600, and 1600–3200 AFU (machine background was typically 100–140 AFU so values below 100 were not obtained), and the data were pooled from 3 separate experiments (mean ± S.E., *n* = 3) similar to the one illustrated in *panels A* and *B. Panels D* and *E* are from a similar experiment except that all cells received Ad ERK2-GFP, and they were also pretreated for 1 or 24 h with 30 μm ERK inhibitor FR180204 (*FR*) or with control medium (*Ctrl.*) as indicated before fixing, staining (for ppERK2), and imaging. The data shown are frequency distribution plots for single cell measures of ERK2 (*D*) and ppERK2 (*E*) in unstimulated cells, and the data are from a single representative experiment with the higher Ad titer. *Panel F* was generated by binning individual cells from both Ad titers (1 and 10 pfu/nl) according to their ERK2-GFP expression level as above. The data were pooled from three separate experiments (mean ± S.E., *n* = 3) similar to the one illustrated in *panels D* and *E*. In addition data are shown for cells pretreated for 24 h with 30 μm compound 328008, a structurally related negative control compound (−*ve*) that does not inhibit ERK.

Negative feedback can influence cell-cell variability irrespective of whether the average response is graded or all-or-nothing. In the first instance we explored this for unstimulated cells where frequency-distribution curves revealed single log ERK and log ppERK peaks and near-Gaussian distribution (not shown). We might expect greater variation of ppERK than of total ERK (due to additional variance in the proportion that is phosphorylated), but the opposite was observed as coefficients of variation (CVs) calculated for single cell measures were higher for ERK (0.030) than for ppERK (0.025), implying that negative feedback restricts variability in ppERK levels. We also used the ERK knockdown/add-back protocol and found that cell-cell variability in ppERK2 levels was again lower than that for ERK2 (compare the spread for the *open circles* in Fig. 2, *A* and *B*, or in *D* and *E*, and see the Fig. 2 legend for CVs). This protocol enables use of mutant reporters (27, 29) so we performed the same experiment using Ad expressing catalytically inactive K52R ERK2 (again as a GFP chimera). Frequency-distribution plots for wild-type ERK2 and K52R ERK2 were indistinguishable (Fig. 2*A*, *open* and *filled circles*) with CVs of ∼0.04, whereas the corresponding plots for ppERK2 revealed lower variability for ERK2 than for K52R ERK2 (Fig. 2*B*, *open* and *filled circles*, for which CVs were 0.028 and 0.05, respectively). Thus, negative feedback mediated by catalytic activity of ERK reduces cell-cell variability in ppERK levels in the face of variability in total ERK levels for unstimulated HeLa cells.

In the experiments above Ad ERK2-GFP was used at 1 pfu/nl to give ERK2 levels close to that of endogenous ERK, but we also performed similar experiments using the Ad at 10 pfu/nl. This caused the expected increase in population-averaged ERK2 expression (∼4-fold) and also increased ERK2 cell-cell variability (CVs were ∼0.04 and 0.12 with 1 and 10 pfu/nl, respectively). This increase in ERK2 variability was associated with increased variation for dual phosphorylation of K52R ERK2 (CVs 0.049 and 0.096, respectively) but not of WT ERK2 (CVs 0.028 and 0.030, respectively), confirming the effect of negative feedback above. The protocol also provides a broad range of ERK2 levels for exploration of relationships between total ERK2 and the system response. Binning the data obtained with both Ad titers according to ERK2 levels and plotting this against ppERK2 revealed a near linear increase in ppERK2 as K52R ERK2 increased to 1000 AFU (Fig. 2*C*), whereas no such increase was seen in cells expressing wild-type ERK2.

As a complementary approach we used a competitive inhibitor of ERK, FR180204, that prevents its catalytic activity but not its Thr-Glu-Tyr phosphorylation (35). In preliminary experiments we used 1 μm PDBu to activate an Egr-1-luciferase reporter and found near maximal inhibition (∼90%) of this ERK-mediated response with 30 μm FR180204 (data not shown; see Ref. 29 for methods). We then used the ERK knockdown/add-back protocol above, treating unstimulated cells for 1 or 24 h with 30 μm FR180204 before imaging for ERK2 and ppERK2. The inhibitor did not measurably influence population average ERK2 levels but increased average ppERK2 from 192 ± 7 to 236 ± 8 AFU (mean ± S.E., *n* = 3, *p* < 0.05). Frequency distribution plots (Fig. 2, *D* and *E*) revealed that FR180204 increased cell-cell variability of ppERK2 levels but not ERK2 levels (as illustrated by the increasing spread of the distributions in Fig. 2*E* but not in 2*D*). We also treated cells for 24 h with a structurally related negative control compound that does not inhibit ERK (328008) and again exploited the broad range of ERK2 levels to define relationships between total ERK2 and ppERK2. Data binning revealed little or no increase in ppERK2 with increasing levels of ERK2 in control cells or in cells treated with the negative control compound (Fig. 2*F*). In contrast, increasing ERK2 was associated with marked increases in ppERK2 in cells exposed for 1 or 24 h to FR180204. The data obtained with chemical inhibition (Fig. 2, *D–F*) are thus similar to those obtained with the catalytically inactive ERK2 mutant (Fig. 2, *A–C*). Together, they confirm earlier work showing that ERK activity is required for negative feedback control of population-averaged ppERK values (29) and, moreover, go on to show that in unstimulated cells, such feedback also controls cell-cell variability in ppERK levels and increases system robustness to changes in ERK levels.

**FIGURE 3.**
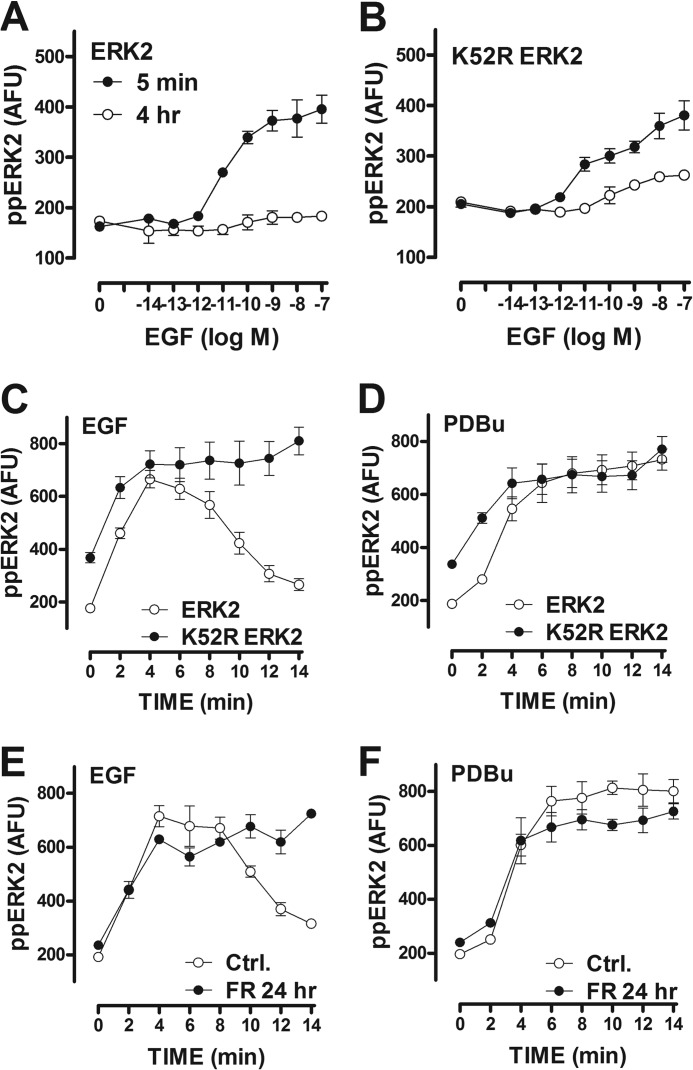
**In stimulated HeLa cells ERK-mediated feedback influences the kinetics of EGF effects on population average ppERK responses and also cell-cell variability in ppERK levels.** *Panels A* and *B*, cells were cultured and transfected with siRNA targeting endogenous ERK1 and ERK2 and transduced with Ad ERK2-GFP or Ad K52R ERK2-GFP before stimulation with EGF for 5 min (*filled circles*) or 4 h (*open circles*) and then stained and imaged (for DAPI and ppERK2). The data are population average ppERK2 levels (mean ± S.E. *n* = 2–6). For each of these experiments we also calculated CVs for the single cell measures, and pooling these revealed that the concentration-dependent effects of EGF on population-averaged ppERK values were paralleled by the effects on cell-cell variability. CV values for ERK2-GFP-expressing cells treated for 5 min with 0, 10^−14^, 10^−13^, 10^−12^, 10^−11^, 10^−10^, 10^−9^, and 10^−7^ m EGF were 0.022, 0.019, 0.024, 0.025, 0.041, 0.058, 0.070, and 0.067, respectively, and this effect was less pronounced in cells stimulated for 4 h with EGF (CV values 0.023, 0.027, 0.023, 0.020, 0.019, 0.025, 0.032, and 0.036 respectively, over the same EGF concentrations). The effect of 5 min of treatment with EGF was also less pronounced in cells expressing K52R-ERK2 (CV values 0.041, 0.050, 0.056, 0.055, 0.059, 0.063, 0.065, and 0.068 for the same EGF concentrations in K52R ERK2-expressing cells), and the mutation did not prevent the time-dependent reduction in effect of EGF on CVs (CV values 0.041, 0.041, 0.041, 0.041, 0.044, 0.046, 0.049, and 0.057 for the same EGF concentrations at 4 h in K52R ERK2-expressing cells). *Panels C* and *D*, cells were treated as above except that they were transduced with ERK2-GFP (*open circles*) or K52R ERK2-GFP (*filled circles*) and were stimulated for 0–14 min with 10 nm EGF (*C*) or 1 μm PDBu (*D*). Population-averaged ppERK2 values are shown (mean ± S.E., *n* = 6). *Panels E* and *F* show population-averaged ppERK2 levels in cells expressing ERK2-GFP and stimulated for the indicated periods using 10 nm EGF (*E*) or 1 μm PDBu (*F*). The cells had been pretreated for 24 h with medium containing 0 (*Ctrl.*) or 30 μm FR180204 to inhibit ERK as indicated (mean ± S.E., *n* = 3).

The fact that that ERK-mediated negative feedback reduces population-averaged ppERK levels in unstimulated cells (Fig. 2) raises the question of whether it also underlies desensitization of ppERK responses in stimulated cells (Fig. 1). To test this, we used the ERK knockdown/add-back protocol, constructing concentration-response curves for EGF at 5 min and 4 h. This revealed clear concentration-dependent increases in ppERK2, and the desensitization was less pronounced in cells with catalytically inactive K52R ERK2 (Fig. 3, *A* and *B*). Physiological EGF concentrations are estimated to be between 0.1 and 10 nm (36), and for each of these concentrations the reduction in ppERK2 occurring between 5 min and 4 h was at least 2-fold higher in cells with ERK2 than in cells with K52R ERK2 (Fig. 3, *A* and *B*). Similar data were obtained in PDBu-stimulated cells (not shown).

These data support a role for ERK-mediated feedback that could be dependent or independent of transcription. To test for the latter we used acute stimulation (2–14 min), and this revealed rapid increases in ppERK with EGF and PDBu in cells with ERK2. The response to EGF was more transient (than that to PDBu) and, importantly, was more sustained in cells expressing K52R ERK2 (Fig. 3, *C* and *D*). Similar experiments were performed with the ERK inhibitor FR180204, and these revealed similar results. Again, the response to EGF was more transient than that to PDBu (in the absence of the inhibitor) but was sustained after 24 h of pretreatment with FR180204 (Fig. 3, *E* and *F*). Interestingly, the response to EGF was also more sustained after only 1 h of pretreatment with FR180204 (*i.e.* the effects of 1 and 24 h FR180204 treatment on response kinetics were indistinguishable; not shown), whereas the effect of FR180204 on the ERK2-ppERK2 relationship was more pronounced at 24 h than at 1 h (Fig. 2*F*). Presumably the FR180204 effect on basal ppERK develops slowly because ERK activity is low in unstimulated cells rather than because FR180204-mediated inhibition of ERK is slow. Together these data reveal that ERK-mediated negative feedback affects population-averaged ppERK responses under short term stimulation conditions (within 10 min with EGF; Fig. 3) and also contributes to the desensitization occurring between 5 min and 4 h (Fig. 3).

**FIGURE 4.**
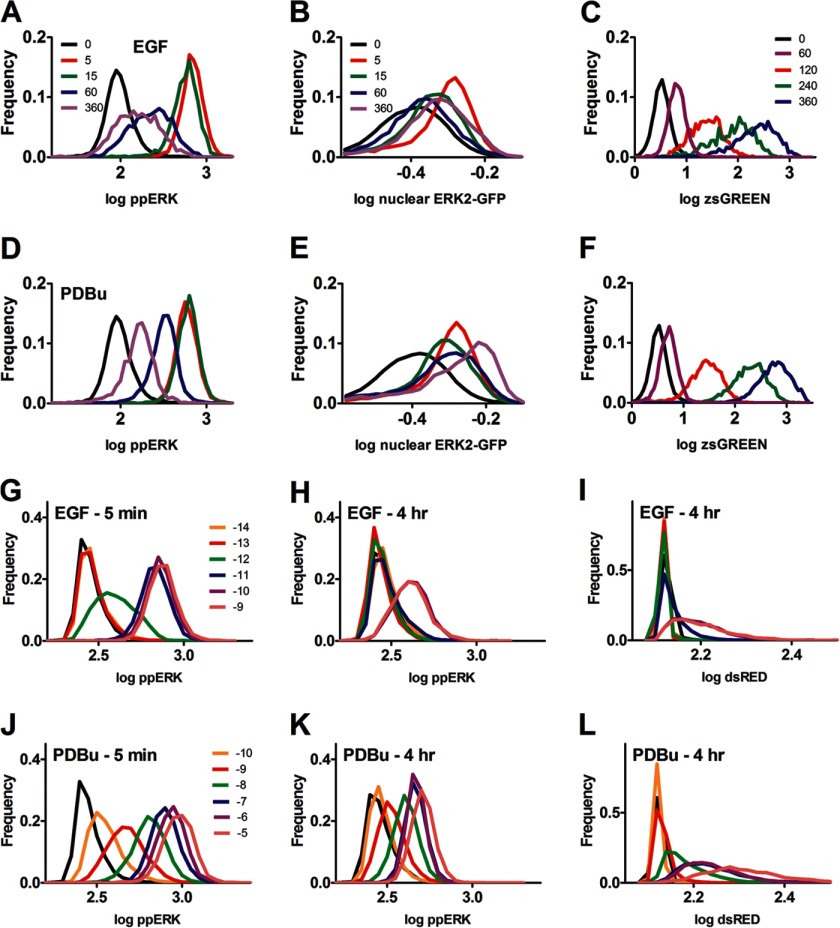
**Frequency-distribution plots reveal unimodal distributions irrespective of the stimulus used, stimulus duration, stimulus concentration, or end point measured.** *Panels A–F*, plots were generated from the single cell data of the time-course experiments shown in Fig. 1, *C–E*. Frequency-distribution plots are shown for log-transformed ppERK (in AFU, *panel A* and *D*), % nuclear ERK2-GFP (*panel B* and *E*) or zsGREEN (in AFU, *panel C* and *F*) in cells stimulated for varied periods with EGF (*A–C*) or PDBu (*D–F*). The legend shows stimulus duration in minutes; in each case the legends in the *top row* apply also to the *panels in the second* row, and several time points are omitted for clarity. The cells for *panels A* and *D* contained endogenous ERK1 and ERK2, whereas those for *panel B* and *E* were transfected with siRNA targeting endogenous ERK1 and ERK2 and transduced with Ad ERK2-GFP so that the proportion of ERK2-GFP in the nucleus could be calculated. *Panels G–L*, plots were generated from the single cell data acquired in the concentration dependence experiments in Fig. 1. The figure shows frequency-distribution plots for log transformed ppERK or dsRED in cells stimulated for 5 min or 4 h as indicated with EGF or PDBu. The legends show log M concentration (*black lines* are for control cells without stimulus). The legends for *G* apply also to *H* and *I*, and those on *J* apply also to *K* and *L*. All cells contained endogenous ERK1 and ERK2. Note that the dsRED values are lower than those for zsGREEN, as the latter is more efficiently imaged, and that the unstimulated values for these reporters differ because machine background values (100–150 AFU) were subtracted from the data in *C* and *F* but not from those in *I* and *L*. For all panels, the plots are each derived from >1000 cells and are from a single representative experiment.

The observation that ERK-mediated feedback influences cell-cell variability in unstimulated cells (Fig. 2) raises the question of whether this is also the case in stimulated cells. To assess this, we constructed frequency-response curves for the single cell measures corresponding to the population-averaged data in Fig. 1 and 3. We suspected that different stimuli might yield all-or-nothing and graded responses and that ERK-mediated negative feedback might alter such behavior, but we found no evidence for this. Indeed, each of the measures (log transformed levels of ppERK or ppERK2, proportion of ERK2 within the nucleus, and levels of Egr-1 zsGREEN or dsRED) showed near Gaussian distributions, and the concentration-dependent effects of EGF and PDBu on these outputs were always graded (Fig. 4). Treatments caused the distributions to shift along the horizontal axes rather than redistributing to or from distinct responsive and unresponsive subpopulations irrespective of whether the cells expressed endogenous ERK or were transduced with wild-type or K52R ERK2 (Fig. 4 and data not shown). Nevertheless, we did observe the effects of stimulation on cell-cell variability, as evidenced by increased spread of frequency-response curves and associated increases in CVs with each of the stimuli and each experimental measure. Notably, the concentration-dependent increase in log ppERK2 caused by 5 min with EGF (Fig. 3*A*) was paralleled by a concentration-dependent increase in CVs for log ppERK2 (see the legend to Fig. 3). Here, it is important to recognize that the variation is normalized to the mean response (*i.e.* cell-cell variability is increased by stimulation rather than simply scaling with the mean response). Moreover, this effect was much less pronounced in cells stimulated for 4 h (see the legend to Fig. 3). Similar data were obtained in PDBu-stimulated cells (not shown). Similar analysis for cells stimulated up to 14 min with EGF or PDBu (Fig. 3, *C* and *D*) revealed that the effects of both stimuli on the CVs of ppERK2 were evident at 2 min and maximal at 6–8 min, although no measurable reduction in ppERK2 CVs occurred between 6 and 14 min irrespective of whether the cells expressed ERK2 or K52R ERK2 (not shown).

The simplest interpretation of these data is that ERK-mediated negative feedback reduces cell-cell variability in ppERK levels under the equilibrium condition of unstimulated cells (Fig. 2) and that this effect is lost when the equilibrium is perturbed by acute stimulation (2–14 min) but becomes re-established due to negative feedback developing between 14 min and 4 h. Moreover, this negative feedback is ERK-mediated, as the reduction in ppERK2 CVs occurring between 5 min and 4 h was much greater in cells expressing WT ERK2 than in cells expressing K52R ERK2 (see the legend to Fig. 3).

##### Total ERK Concentration and Response Kinetics

The demonstration that ERK-mediated feedback influences system robustness to changes in ERK concentration in unstimulated cells (Fig. 2) raises the question of whether it also does so after stimulation. In a recent study (16) siRNA knockdown of ERK 1 and/or 2 was used to explore relationships between ERK and ppERK levels (in steady-state conditions of chronic stimulation). It was argued that these measures would be directly proportional without robustness and shown that ERK-mediated negative feedback conferred partial robustness on the system, as indicated by a saturating (near hyperbolic) relationship between ERK and ppERK levels.

**FIGURE 5.**
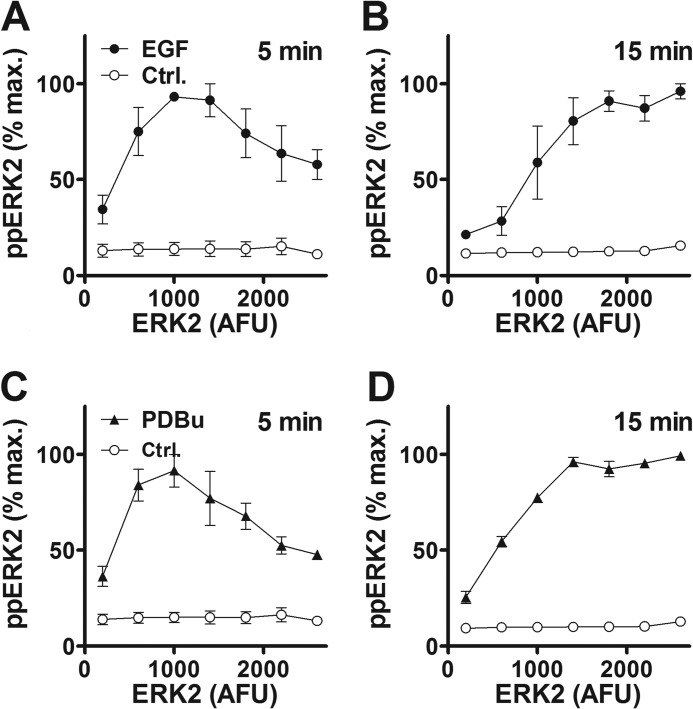
**Binning HeLa cells according to ERK2 expression level reveals a time-dependent switch from a bell-shaped to a monotonic ERK2-ppERK2 relationship.** Cells were transfected with siRNA targeting endogenous ERK1 and ERK2 and transduced with Ad ERK2-GFP before treatment for 5 or 15 min with 10 nm EGF (*panels A* and *B*, *filled circles*) or 1 μm PDBu (*panels C* and *D*, *filled triangles*) or without stimulus (*Ctrl.*, *open circles*). Individual cells were binned according to ERK2-GFP expression, and mean ppERK2 level was calculated for each bin. The figures show ppERK2 levels plotted against bin center and are pooled from three experiments (mean ± S.E., *n* = 3).

**FIGURE 6.**
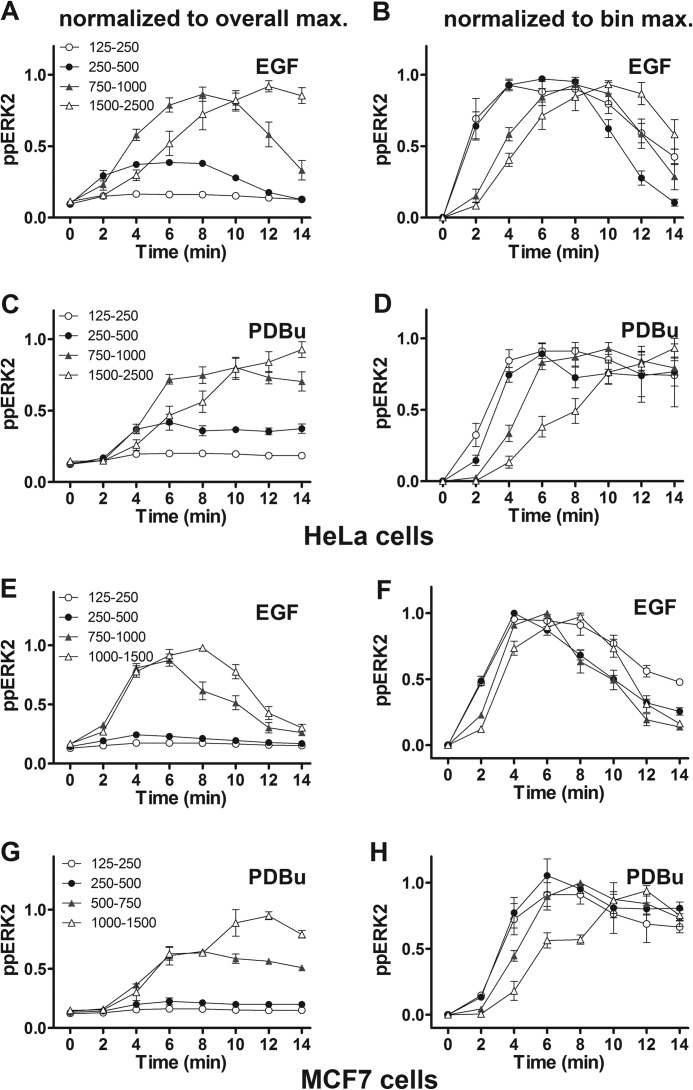
**ppERK2 activation develops more rapidly at low ERK2 levels in HeLa and MCF7 cells.** HeLa cells (*panels A–D*) or MCF7 cells (*panels E–H*) were treated as described under Fig. 5, with 10 nm EGF (*A*, *B*, *E*, and *F*) or 1 μm PDBu (*C*, *D*, *G*, and *H*) as indicated, except that the stimulus was for 0–14 min. After imaging, individual cells were binned for calculation of mean ppERK2 level for the indicated ERK2-GFP bins (values in AFU). The *left panels* show ppERK levels normalized to the maximum level with the appropriate stimulus irrespective of ERK2-GFP bin, whereas in the *right panels* levels were normalized to the maximum ppERK level in any given ERK2-GFP bin, to emphasize differences in amplitude and kinetics, respectively. The legends in the left hand panels also apply to the corresponding right hand panel and the data shown are pooled from 3 separate experiments (mean ± S.E., *n* = 3).

We exploited the broad range of ERK2 levels in our Ad ERK2-GFP add-back protocol (Fig. 2) by sorting the data from individual cells into bins according to ERK2 expression level and plotting the mean ppERK level per bin (Fig. 5). From the earlier study (16) we anticipated that ERK and average ppERK levels might initially be directly proportional and that this relationship might be lost with the time-dependent development of ERK-mediated negative feedback, but this was not the case. Remarkably, the relationship between ERK and ppERK levels was initially bell-shaped (5 min after stimulation) with maximal ppERK2 levels obtained at submaximal ERK2 levels using maximally effective concentrations of EGF or PDBu (Fig. 5, *A* and *C*). In contrast, monotonic relationships (with maximal ppERK2 at maximal ERK2 levels) were seen for both stimuli at 15 min (Fig. 5, *B* and *D*) and thereafter (0.5, 1, 2, and 4 h; data not shown). The implication that ppERK response kinetics are dependent upon ERK concentration was tested by assessing the time-course for acute activation in HeLa cells binned according to ERK2-GFP expression level. As shown (Fig. 6, *A–D*), increasing ERK2 expression not only increased maximal responses to EGF and PDBu but also slowed the responses. The latter is most obvious with data normalized according to the maximum response for each ERK2 bin (Fig. 6, *B* and *D*). The time required to reach the half-maximal response increased (from 1.68 ± 0.37 to 6.24 ± 0.29 min in EGF-stimulated cells and from 2.65 ± 0.23 to 7.75 ± 0.30 min in PDBu-stimulated cells) as ERK2 levels increased from bins of 125–250 to 1500–2500 AFU. Most importantly, however, the bell-shaped relationship between ERK2 and ppERK2 levels was again seen at early time points with maximal ppERK2 levels at submaximal ERK2 levels (bins of 250–500 and 750–1000 AFU for EGF and PDBu at 2 min), whereas the relationships were again monotonic at later time points (maximal ppERK2 at maximal ERK2 levels occurred at 12 min for both stimuli). Log ppERK2 frequency-distribution plots were also generated (data not binned according to ERK2), and these were unimodal irrespective of the time point (2 or 12 min) or stimulus used (not shown). Similarly, when EGF dose-response curves were constructed at 2 and 12 min, log ppERK2 frequency-distribution plots were unimodal and responses were graded with all concentrations (0 or 10^−12^ to 10^−7^ m EGF) at both times (data not shown).

We were interested to know whether the dependence of ppERK response kinetics on ERK levels was a specific feature of HeLa cells and, therefore, performed similar experiments in MCF7 breast cancer cells. Again, we used the ERK knockdown/add-back protocol, stimulated cells for up to 14 min with EGF or PDBu, and quantified ERK2 and ppERK2 levels in individual cells before binning according to ERK2 level. The data obtained were qualitatively very similar to those with HeLa cells in that responses were not only greater at higher ERK2 levels (Fig. 6, *E* and *G*) but were also slower at higher ERK levels, as seen in data normalized to the internal control maximal response in any given ERK2 bin (Fig. 6, *F* and *H*). As with the HeLa cell data, the time required to reach the half-maximal response increased with increasing ERK2 levels in MCF7 cells (from 2.05 ± 0.05 to 3.53 ± 0.11 min with EGF at 125–250 and 1000–1500 AFU and from 3.55 ± 0.16 to 5.90 ± 0.22 with PDBu and the same ERK2 bins). Moreover, the relationship between ERK2 level and ppERK2 levels was bell-shaped at 2–4 min (maximal responses in ERK2 bins of 250–500 or 500–750 AFU for both stimuli and both cell types) and had switched to an increasing monotonic relationship (maximal responses in the highest ERK2 bin) by 12 min for both stimuli and both cell types (not shown).

**FIGURE 7.**
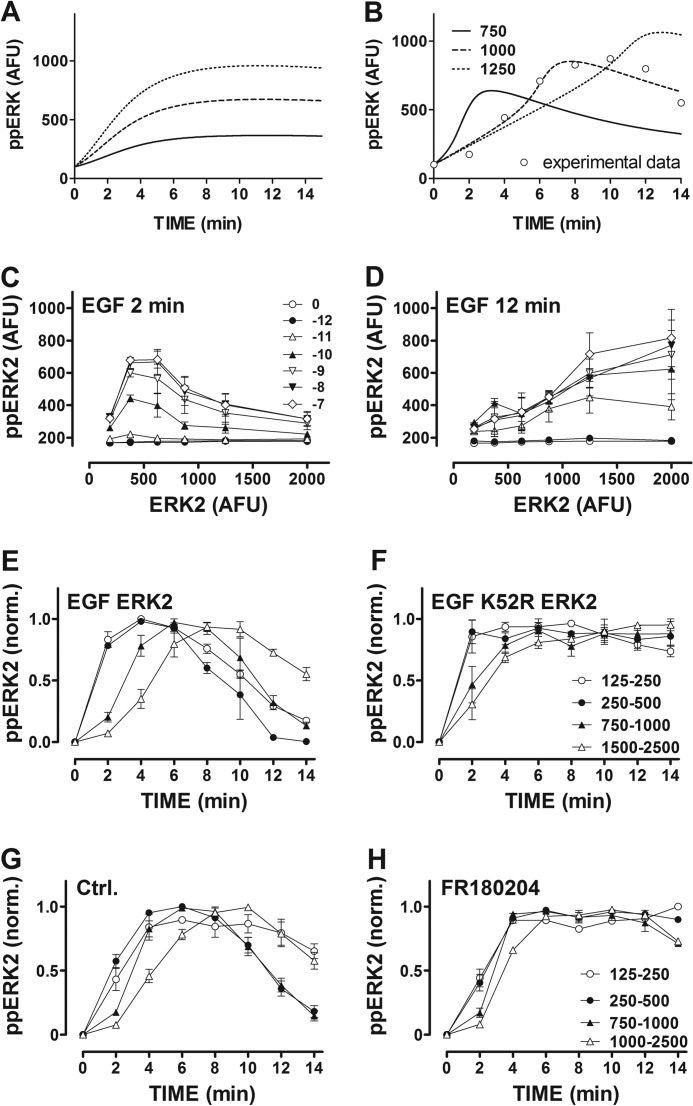
**Dependence of ppERK response kinetics on ERK level can be mathematically modeled assuming distributive (but not processive) activation and occurs at a wide range of EGF concentrations and is not dependent on ERK-mediated feedback.** *Panels A* and *B*, shown are whole cell ppERK levels, as predicted by mathematical modeling of ERK activation assuming either processive (*A*) or distributive (*B*) dual phosphorylation of ERK (models are described in the supplemental data). In each case ERK concentration was set at low, intermediate, or high using arbitrary values of 750, 1000, and 1250 AFU, respectively (as indicated). Experimental data (EGF stimulation at 750–1000 AFU ERK2-GFP) are superimposed in *panel B* (*open circles*). *Panels C* and *D*, cells transfected with siRNA targeting ERK and then transduced with Ad ERK2-GFP before stimulation for 2 or 12 min with EGF as shown. Mean ppERK2 values are plotted against ERK2-GFP bin center (mean ± S.E., *n* = 3, the legend in *C* applies also to *D*). *Panels E* and *F*, cells were transfected with siRNA targeting ERK and then transduced with Ad ERK2-GFP or K52R ERK2-GFP before stimulation for 0 or 2–14 min with 10 nm EGF. Mean ppERK2 values are shown against time for the indicated ERK2-GFP bin centers (mean ± S.E., *n* = 3, legend in *F* applies also to *E*) and normalized (*norm.*) to the maximum ppERK level in any given ERK2-GFP bin. *Panels G* and *H*, cells were transfected with siRNA targeting ERK, transduced with Ad ERK2-GFP, and pretreated for 24 h with 0 (*Ctrl.*) or 30 μm FR180204 before stimulation for 0 or 2–14 min with 10 nm EGF. Mean ppERK2 values are shown against time for the indicated ERK2-GFP bin centers (mean ± S.E., *n* = 3, legend in *H* applies also to *G*) and normalized (*norm.*) to the maximum ppERK level in any given ERK2-GFP bin.

Early biochemical studies showed that the activating dual phosphorylation of ERK is distributive (24). However, its activation in intact cells may be (pseudo)processive due to scaffolding or molecular crowding (26). We used mathematical modeling to address whether our data could best be explained by distributive or processive activation. We considered two distinct descriptions of the ERK activation pathway, both based on the law of mass action, but applied to either processive or distributive double phosphorylation (for ERK activation) based on Markevich *et al.* (17). We used gradient-based minimization procedures implemented in MATLAB (Mathworks, Natick, MA) to fit the two models to the HeLa cell experimental data at medium ERK concentrations (for an example see Fig. 7*B* with ERK at 750–1000 AFU). This allowed estimation of parameters and initial conditions for the two models using the same experimental data points (details in the supplemental data). Having calibrated the models we then varied only the total ERK concentration to simulate low, intermediate, and high ERK levels. The two models predict markedly different responses (Fig. 7). The processive activation model predicts that increasing ERK concentration will simply increase the ppERK response amplitude (Fig. 7*A*), whereas the distributive model predicts that increasing ERK concentration will also slow response onset (Fig. 7*B*). Both models predict a monotonic relationship between ERK concentration and ppERK level at later time points (12 and 14 min), whereas only the distributive model predicts an initial non-monotonic relationship with maximal activation at sub-maximal ERK concentration (Fig. 7, *A* and *B*).

The key finding of the mathematical modeling is that only the distributive model accurately mirrors the wet laboratory data by predicting dependence of response kinetics on ERK concentration and a switch in the relationship between ERK and ppERK (from non-monotonic to monotonic) during the first 15 min of stimulation. The model predicted that this switch would occur at a range of inputs. When input intensity was varied (by setting the active MEK to 2.5, 25, 50, or 100 arbitrary units) the model predicted bell-shaped ERK-ppERK relationships at 2 min and monotonic relationships at 12 min (not shown). We tested this experimentally by constructing EGF concentration-response curves at 2 min and at 12 min in HeLa cells. For each EGF concentration and time point, data were binned according to ERK2 level, and non-monotonic ERK2-ppERK2 relationships were seen at 2 min (but not at 12 min) for EGF concentrations from 10^−12^ to 10^−7^ m (Fig. 7, *C* and *D*). Importantly, the distributive model predicts occurrence of the switch in the absence of ERK-mediated negative feedback, so we tested for this by repeating the experiments in Fig. 6 using K52R ERK2-GFP. As expected, EGF rapidly increased ppERK2 levels in cells expressing ERK2-GFP, with the effects being slower in onset at higher ERK2-GFP levels (Fig. 7*E*). EGF also increased ppERK2 levels in cells expressing K52R ERK2-GFP, and again the responses were slower in onset in cell bins expressing higher levels of ERK2-GFP (Fig. 7*F*). Similar data were obtained in PDBu-stimulated cells, and as above there was a switch with the relationship between ERK2-GFP and ppERK level being non-monotonic at 2 min (maximal responses in 250–500 or 500–750 ERK2 bins) but monotonic at 12 min (maximal responses in 1000–1500 ERK2 bins) for both stimuli. We also tested these relationships in cells treated for 1 h with or without the ERK inhibitor (FR180204) before stimulation with EGF (*i.e.* by binning the data shown as population averages in Fig. 3, *E* and *F*) and found that the inhibitor had similar effects to the K52R ERK2 mutation (compare Fig. 7, *G* and *H* with *E* and *F*). Most importantly, the fact that the switch occurred in K52R ERK2-expressing cells (Fig. 7, *E* and *F*) and in the presence of the ERK inhibitor (Fig. 7, *G* and *H*) implies that this time-dependent change in the relationship between ERK and ppERK levels is not due to ERK-mediated negative feedback.

## DISCUSSION

**FIGURE 8.**
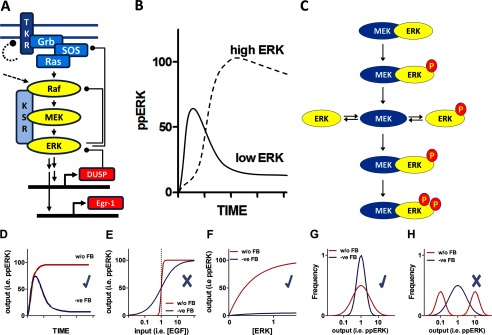
**Summary of system architecture and main findings.** *Panel A* illustrates the ERK cascade with a tyrosine kinase receptor (*TKR*) acting via adapters and effectors (Grb, SOS) to stimulate Ras (H-Ras, K-Ras, or N-Ras). Ras then activates Raf (A-Raf, B-Raf, or C-Raf) causing activating phosphorylation of MEK (MEK1 or MEK2) that in turn catalyzes dual phosphorylation of ERK (ERK1 or -2). Alternative mechanisms include activation of Raf by PKC (*dotted arrow*). Transcription-independent and transcription-dependent (dual specificity phosphatase-mediated) negative feedback pathways are also shown as are upstream receptor-specific feedback mechanisms (*curved dotted line*). Our most important finding is that the ERK expression level influences ppERK response kinetics as well as amplitude (*panel B*) such that maximal activation initially occurs at the submaximal ERK level and then switches to an increasing monotonic relationship (maximal activation at maximal ERK level) within 12 min for each of the cell lines studied. This is apparently not-dependent on ERK-mediated negative feedback (as it is evident in cells expressing catalytically inactive ERK2) and could be modeled assuming distributive activation as illustrated by the schematic in *panel C*. Also illustrated (*lower panels*) are some of the system features potentially regulated by negative (-*ve*) feedback (*FB*). These include population-averaged response kinetics (*D*), the relationship between stimulus concentration (*E*) or ERK concentration (*F*) and system output, cell-cell variability in ppERK levels (*G*), and bimodality as opposed to unimodality of ppERK distributions (*H*). In our HeLa cell model, ERK-mediated negative feedback shapes population-averaged ppERK responses (within 10 min in EGF-stimulated cells as well as between 5 min and 4 h for EGF and PDBu-stimulated cells) (*check* in *D*). It does not influence system sensitivity to EGF or PDBu concentration (*cross* in *E*) but does provide robustness to changes in ERK level in unstimulated cells (*check* in *F*). It also reduces cell-cell variability in ppERK levels in unstimulated cells (*check* in *G*) but does not convert a bimodal distribution to a unimodal one (*cross* in *panel H*). Bimodal distributions were not seen for any of the conditions used or end-points measured.

Many extracellular signals act via the Raf/MEK/ERK cascade in which response amplitude, kinetics, cell-cell variability, sensitivity, and system robustness are fundamental attributes that determine effects of ERK on cell fate. The system is subject to several forms of negative regulation with different mechanisms and time scales (Fig. 8). Such feedback is important for control of cell fate, but most work in this field focuses on chronic (long term) stimulation, with much less known about acute stimulation. A principal aim of this study was to determine how ERK-mediated feedback shapes ERK signaling in cells acutely stimulated with EGF (ErbB1 activation) and PDBu (PKC activation). We used a HeLa cell model in which these stimuli cause more-or-less transient, activating phosphorylation and nuclear translocation of ERK (Fig. 1) and were particularly interested in the role that ERK-mediated feedback plays in the non-equilibrium conditions of acute stimulation. Our data reveal a system in which ERK-mediated negative feedback has major effects in unstimulated cells and that such effects are less evident with short term activation and then recover with continued stimulation.

By expressing catalytically inactive K52R ERK2 (as a GFP fusion) or by chemically inhibiting ERK, we found in unstimulated cells that ERK-mediated negative feedback reduces not only population-averaged ppERK2 levels (Figs. 2 and 3) but also variability in ppERK2 levels across cells (Fig. 2) as well as increasing system robustness to changes in total ERK levels (Fig. 2). Similarly, in acutely stimulated cells, ERK-mediated negative feedback contributes to the reduction in average ppERK levels and the associated reduction in the variability (CVs) of ppERK that occurs between 5 min and 4 h (Fig. 3). Interestingly, ERK-mediated negative feedback rapidly (within 10 min) influenced population-averaged ppERK levels (Fig. 3), but this rapid (transcription-independent) effect was not associated with reduced cell-cell variability in the same timeframe (not shown). Moreover, concentration-response curves for EGF and PDBu effects on ppERK (population average responses) at 5 min and 4 h (Fig. 1) had indistinguishable Hill coefficients, arguing against a change in sensitivity of mean ppERK levels to input intensity as a result of negative feedback. Thus, in stimulated cells we saw relatively rapid ERK-mediated feedback effects on ppERK levels (within 10 min, Fig. 3), slower effects on ppERK variability (within 4 h, see the legend to Fig. 3), and no effect on response sensitivity (within 4 h, Fig. 1). These findings are summarized in Fig. 8).

We found no evidence for negative feedback influencing whether the ppERK response shows graded or all-or-nothing behavior at the population-average level. Furthermore, frequency-distribution curves for populations of single cells (Fig. 4) consistently revealed unimodal responses irrespective of the type and concentration of stimulus used, the stimulus duration, the response measured (ppERK levels, nuclear accumulation of ERK, ERK-driven transcription), and whether or not ERK-mediated feedback was permitted (data not shown). A recent report (37) attributed the bimodal ppERK responses seen in EGF-stimulated HEK293 cells to cell-cell variability in Ras activation and pathway design that incorporates negative feedback rather than to response ultrasensitivity or bistability as often assumed. In this regard it is of interest that we saw only unimodal ppERK responses when ERK expression variability was increased (by varying Ad ERK2 titer). Thus although negative feedback and/or cell-cell variability in protein levels have the potential to influence whether ppERK responses are all-or-nothing at the single cell level and hence bimodal, we saw no bimodality in the HeLa cell system used here (as summarized in Fig. 8).

As noted above, the time-dependent reduction in ppERK2 levels was lower in cells expressing K52R ERK2 (than in cells with ERK2), but the desensitization was not completely prevented by the mutation. This implies that additional mechanisms contribute to the observed adaptation (Fig. 3), and stimulus-induced, down-regulation of ErbB1 (36) likely contributes to desensitization of the EGF effect. Indeed, mathematical modeling has revealed receptor internalization as a major determinant of EGF signal termination (38), and this is consistent with our observed reduction in EGF efficacy and potency that occurs between 5 min and 4 h (Fig. 1). This clearly influences input-output behavior, so it is worth stressing that our observation that there is no reduction in response sensitivity between 5 min and 4 h is based on the lack of measurable change in Hill coefficient with either stimulus (Fig. 1).

We also used cell binning to define relationships between levels of total ERK and ppERK (*i.e.* to explore “concentration robustness” of the signaling system (39)). Our work is related to a previous study (16) where ERK expression was manipulated with inhibitory RNAs targeting ERK1 and/or ERK2, and it was found that ERK-mediated negative feedback increased robustness to total ERK, converting a linear relationship between ERK and ppERK levels to a hyperbolic one. In contrast to this work, we focused on the behavior under acute stimulation (as opposed to chronic activation by mutation of Ras or Raf). Our data revealed a complex and initially surprising relationship. For both stimuli the relationship between ERK and ppERK levels was bell-shaped at 5 min and approximately hyperbolic at later time points (Fig. 5). To explore further, we focused on early time points (0–14 min), and again, the responses to both stimuli were slower at higher ERK levels, as revealed by the longer time taken to achieve the half-maximal ppERK response (Fig. 6). The switch from bell-shaped ERK2-ppERK2 input-output behavior at 2 or 4 min to increasing monotonic relationships at 12 min was again observed (Fig. 6). Importantly, this behavior was not restricted to HeLa cells as ppERK response amplitude and kinetics were also dependent on ERK expression levels in MCF7 (breast cancer) cells (Fig. 6). Despite inevitable quantitative differences between responses in these cells, the novel and unexpected observation of a switch from an initial bell-shaped ERK2-ppERK2 relationship at 2–4 min to an increasing monotonic one at 12 min was made with both cell lines tested (Fig. 6).

Our key observation was that maximal ERK activation occurs initially at a submaximal ERK concentration, but the mechanisms underlying this effect remain unclear. When ERK activation is distributive, ERK binds to MEK and is then monophosphorylated and released before rebinding to facilitate the second phosphorylation in the Thr-Glu-Tyr loop. Consequently, non-phosphorylated ERK may compete with monophosphorylated ERK for binding to MEK and thereby inhibit the second phosphorylation, which is needed for activation as well as for detection by the ppERK antibodies used here (Fig. 8). Such competition is consistent with the key observation above. In early *in vitro* studies increasing ERK concentration actually reduced ppERK concentration in kinase assays with semi-purified MEK and ERK (24). The alternative scenario is that of processive dual phosphorylation in which a substrate binds a kinase and is dual-phosphorylated without release and rebinding. Accordingly, we used a mathematical modeling approach and found that our data could be accurately predicted by modeling activation as distributive (but not as processive) (Fig. 7).

Distributive activation provides the simplest explanation for our data (Fig. 8). However, a recent study concluded that ERK activation is (pseudo)processive as a consequence of “molecular crowding,” providing a possible explanation for the observed graded responses in mammalian cells (26). By contrast, the early mathematical modeling of distributive activation in the absence of negative feedback predicted an ultrasensitive ppERK response of the cascade itself, whereas we did not observe such non-graded responses at any time point. Interestingly, our findings of graded responses both in the presence and absence of negative feedback are similar to those for the Fus3 response in yeast (40). EGF concentration curves constructed at 5 min (Fig. 1, see also Ref. 26) had a Hill coefficient of >1 (demonstrating sensitivity greater than expected for simple Michaelis-Menten kinetics), but the Hill coefficient for PDBu was <1 under the same conditions, suggesting that these measures reflect upstream features rather than sensitivity of the Raf/MEK/ERK cascade itself. It is also noteworthy that ERK stimulation is typically associated with translocation of the KSR scaffold from the cytoplasm to plasma membrane, raising the intriguing possibility that there could be a time-dependent shift from unscaffolded to scaffolded ERK activation (41) and an associated shift from distributive to (pseudo)processive activation. To our knowledge this scenario has not yet been modeled mathematically or tested experimentally. Finally, it is important to recognize that more recent mathematical studies highlight the potential for multisite phosphorylation to influence system thresholding without generating ultrasensitive behavior (42). In the context of cell fate, this implies that Raf/MEK/ERK thresholding could determine whether or not individual cells commit to all-or-nothing fate decisions without, itself, dictating their binary nature.

Another possible explanation for our data, but one we are able essentially to rule out, is that overexpression of ERK in our system might saturate KSR1 scaffolds (thereby causing distributive activation) *in vivo*. Published estimates of cellular ERK concentrations range from ∼0.25 to 2.1 μm in HeLa, PC12, CHO, and COS-7 cells (43). However, our data reveal considerable cell-cell variability with the mean concentration being ∼5-fold lower in the lowest 5 percentile than in the highest 5 percentile, suggesting that the majority of cells (95%) would have endogenous ERK concentrations between 0.12 and 4.7 μm. The mean ERK2-GFP concentration is comparable to endogenous ERK levels with the low Ad titer knockdown/add-back protocol (29, 33), so we used this titer to determine ERK concentrations from the ERK2-GFP AFU measures, obtaining estimates of 0.3, 0.8, 1.8, 2.8, 4.1, and 7.1 μm (for the ERK2-GFP bins used in Fig. 5). Thus, the vast majority of cells used for this study (those in bins of up to 1500 AFU) expressed ERK2-GFP at concentrations found for endogenous ERK in commonly used cell lines. Most importantly, the initial non-monotonic ERK2-ppERK relationship occurs with physiologically relevant EGF concentrations (36) and within the range of ERK2-GFP concentrations encountered with endogenous ERKs (Fig. 5).

Irrespective of the exact mechanisms, our data and the associated mathematical modeling are suggestive of distributive activation within the first 5 min of stimulation with EGF or PDBu. The distributive activation model that most closely fits our experimental data predicted that the switch from an initial non-monotonic response (at 2 min) to a monotonic response (by 12 min) would occur for a range of upstream input intensities (modeled as varied MEK activity), and consistent with this, we found that the switch occurs at a broad range of EGF concentrations (Fig. 7). Importantly, the distributive activation model does not include ERK-mediated negative feedback, so we also tested this using catalytically inactive ERK and by ERK inhibition. This revealed that the initial maximal activation of ERK at the submaximal ERK expression level also occurred in cells expressing the K52R mutant and in cells treated with FR180204 (Fig. 7). These data clearly argue that the switch is not dependent upon ERK-mediated negative feedback despite the fact that such feedback does occur in this time frame (Fig. 3). It is also not dependent upon upstream ErbB1-specific negative feedback, as it is also seen with ErbB1-independent ERK activation by PDBu in both cell lines (Figs. 5 and 6).

An intriguing aspect of the observed change in the relationship between ERK and ppERK levels is that it provides the potential for the control or “gating” of the response by the interplay of the ERK expression level and the stimulus duration. For example, in our HeLa cell system, a 2-min stimulation with EGF caused little or no ERK phosphorylation at the highest ERK expression levels (1500–2500 AFU) and more pronounced phosphorylation at lower levels (250–500 AFU) (Fig. 5). The opposite is true with a 14-min stimulation, where the amount of ppERK positively correlates with ERK expression level. Similarly, at intermediate ERK levels (750–1000 AFU), 2- and 14-min stimulations, both, result in less phosphorylation compared with that obtained during the intervening time points. Here, it should be noted that although much work on ERK signaling explores equilibrium situations approached with chronic stimulation (*i.e.* long term exposure to serum, growth factors, or activating mutations), non-equilibrium activation is very likely to be important physiologically. Examples include smooth muscle cells where intrinsic Ca^2+^ oscillations have the potential to drive ERK activation (44, 45) or the neuroendocrine system where hormones acting via G-protein-coupled receptors to activate ERK are secreted in brief pulses. Gonadotropin-releasing hormone (GnRH), for example, can be secreted in pulses of a few minutes duration at intervals of 30–120 min. Each GnRH pulse drives a pulse of PKC-mediated ERK activation, and GnRH receptor-mediated ERK activation is essential for central control of reproduction (46, 47). The data shown here suggest that the concentration of ERK in the target cells could determine whether or not they respond to a GnRH pulse and could also influence the rate of response termination after stimulus removal. Cells expressing a high level of ERK would be expected not to have time to respond appreciably before the pulse terminates, providing a mechanism for gating of the response by the level of ERK. In this scenario, cell-cell variation in the level of ERK expression could dictate the proportion of cells responding to pulsatile GnRH stimulation, a feature thought to be important for GnRH signaling (48, 49). Thus, although we are not currently aware of specific physiological scenarios in which ErbB1 occupancy changes rapidly, a surprising prediction of our work is that increasing ERK levels could actually reduce ERK activation under physiologically relevant stimulus durations for some stimuli.

In summary, we have used automated cell imaging to explore the role of ERK-mediated negative feedback in shaping ERK signaling in HeLa cells and have found clear evidence that ERK-dependent feedback influences ppERK response kinetics, variability, and robustness in unstimulated cells just as it does in chronically stimulated cells (Fig. 8). In acutely stimulated cells, we observed reduction by ERK-mediated negative feedback of average ppERK levels and cell-cell variation in these levels but did not see any effect of negative feedback on the sensitivity of ERK activation to stimulus concentration. When we considered robustness of the signaling system, we saw an unexpected, bell-shaped relationship between total ERK and ppERK levels at 2–5 min that switched to an increasing monotonic one within 12 min of stimulation. This novel behavior occurred in two cell types (HeLa and MCF7 cells) in the presence and absence of negative feedback and was predicted by mathematical models incorporating distributive activation in acutely stimulated cells but not by those with processive activation. The rapid switch makes response kinetics dependent on ERK level and provides a gating mechanism by which variation in ERK expression influences ERK responses under acute stimulation.

## Footnotes

3 The abbreviations used are:

ppERK: dual phosphorylated ERK
PDBu: phorbol 12,13-dibutyrate
Ad: adenovirus
AFU: arbitrary fluorescence units
CV: coefficient of variation
GnRH: gonadotropin-releasing hormone.
